# Inactivation mechanism of N61S mutant of human FMO3 towards trimethylamine

**DOI:** 10.1038/s41598-017-15224-9

**Published:** 2017-11-07

**Authors:** Chongliang Gao, Gianluca Catucci, Silvia Castrignanò, Gianfranco Gilardi, Sheila J. Sadeghi

**Affiliations:** Department of Life Sciences and Systems Biology, University of Torino, Turin, Italy

## Abstract

Human flavin-containing monooxygenase 3 (hFMO3) catalyses the oxygenation of a wide variety of compounds including drugs as well as dietary compounds. It is the major hepatic enzyme involved in the production of the N-oxide of trimethylamine (TMAO) and clinical studies have uncovered a striking correlation between plasma TMAO concentration and cardiovascular disease. Certain mutations within the hFMO3 gene cause defective trimethylamine (TMA) N-oxygenation leading to trimethylaminuria (TMAU) also known as fish-odour syndrome. In this paper, the inactivation mechanism of a TMAU-causing polymorphic variant, N61S, is investigated. Transient kinetic experiments show that this variant has a > 170-fold lower NADPH binding affinity than the wild type. Thermodynamic and spectroscopic experiments reveal that the poor NADP^+^ binding affinity accelerates the C4a-hydroperoxyFAD intermediate decay, responsible for an unfavourable oxygen transfer to the substrate. Steady-state kinetic experiments show significantly decreased N61S catalytic activity towards other substrates; methimazole, benzydamine and tamoxifen. The *in vitro* data are corroborated by *in silico* data where compared to the wild type enzyme, a hydrogen bond required for the stabilisation of the flavin intermediate is lacking. Taken together, the data presented reveal the molecular basis for the loss of function observed in N61S mutant.

## Introduction

Human hepatic flavin-containing monooxygenase isoform 3 (hFMO3) catalyses the oxygenation of a wide variety of nitrogen- and sulphur-containing chemicals including drugs as well as dietary compounds resulting in polar metabolites which are readily excreted^1,2^. Previous studies have indicated that the FMO-mediated oxygenation reaction can be separated into two sequential reactions, the reductive and re-oxidative half-reactions^3–5^. In the reductive half-reaction, the FAD cofactor is first reduced through a hydride ion transfer from the nicotinamide C4 atom of NADPH to the flavin N5 atom which subsequently reacts with molecular oxygen to yield the C4a-hydroperooxyFAD intermediate^3–5^. In the presence of substrate, an oxygen atom is transferred from this intermediate to the substrate to generate an oxygenated product and yield a second intermediate C4a-hydroxyFAD. After losing the second oxygen atom and forming a water molecule, this intermediate reverts back to the oxidized FAD. One of the most important features of this catalytic cycle is the fact that after the first step of reduction by NADPH, the NADP^+^ remains bound to the FAD which is essential to protect the C4a-hydroperooxyFAD intermediate from decaying until a substrate gains access to the active site^3–5^.

Human FMO3 has long been known to be the major hepatic enzyme involved in the conversion of trimethylamine (TMA) to trimethylamine N-oxide (TMAO) metabolite^6,7^. TMA is derived from fish as well as the degradation of dietary choline and carnitine by gut bacteria^8^. Clinical studies have uncovered a striking correlation between TMAO and cardiovascular disease with inhibition of TMAO production put forward as a treatment for atherosclerosis^8–10^. However, naturally occurring mutations within the hFMO3 gene also result in lower TMAO production and lead to trimethylaminuria (also known as fish odour syndrome)^11^. Trimethylaminuria is associated with lack of hFMO3 N-oxygenation activity resulting in large amounts of TMA being excreted^12,13^.

To date, more than 18 mutations associated with trimethylaminuria have been identified^14,15^. Of these, the naturally occurring mutation N61S abolishing the N-oxygenation capacity of hFMO3^12^ is of particular interest due to its highly conservative and possible importance in oxygen transfer as reported before^16–18^. In spite of growing knowledge of genetic polymorphisms of hFMO3 and specifically those associated with trimethylaminuria, the molecular mechanisms underlying these loss-of-function genetic variants is still unclear due to lack of detailed structural information for hFMO3 as well as availability of pure and holo-protein. To tackle these problems, we have previously constructed a homology structural model of hFMO3^19^ showing that the protein possesses a small NADP^+^-binding domain and a large FAD-binding domain. In addition, we have also devised protocols for purification of the holo-hFMO3^20,21^.

The aim of this work is to study the inactivation mechanism of hFMO3 in the N-oxygenation of TMA. Kinetic parameters of reductive and re-oxidative half-reactions were determined spectroscopically by stopped-flow and the binding properties of NADP^+^ to hFMO3 were characterized by isothermal titration calorimetry (ITC), circular dichroism (CD), differential scanning calorimetry (DSC) and trypsin digestion. In addition, steady-state kinetic parameters in the presence of different substrates were also determined. The experimental results show that N61S mutation renders hFMO3 inefficient in using NADPH for flavin reduction. Furthermore, poor NADP^+^ binding affinity of the mutant leads to an unstable C4a-hydroperoxyFAD intermediate, abolishing or decreasing the catalytic efficiency of hFMO3 towards its substrates.

## Results

The N61S variant of hFMO3 was expressed in *E*. *coli* and purified by Ni-affinity chromatography by protocols previously described for the wild type enzyme^20,21^, resulting in a yield of 11 mg of the purified protein. The different steps of the hFMO3 catalytic cycle were analysed with the purified wild type and N61S variant in order to determine parameters affected by the mutation known to lead to an inactive enzyme incapable of metabolising TMA and causing the rare disease, TMAU^11–13^.

### Transient kinetics of reductive and re-oxidative half-reactions

The hFMO3 catalytic cycle contains reductive and re-oxidative half-reactions^4,5^. In the first step, the FAD cofactor is reduced by NADPH. The rate of this reductive half-reaction was determined by following the anoxic reduction of FAD at 450 nm by varying the NADPH concentration. Compared to WT-hFMO3, the N61S variant shows poor NADPH binding affinity with a calculated *K*_d_ of 51.8 μM versus 0.3 μM for the wild type (Table 1). When the N61S reductive half-reactions were carried out under the same conditions as those of the wild type, no reduction was observed and therefore the concentration of NADPH had to be increased 10 folds. This is consistent with the poor NADPH binding affinity. With this 10 folds increase in NADPH concentration, a higher k_red_ for the N61S variant was measured (Table 1).

**Table 1:** Kinetic parameters of reductive and re-oxidative half-reactions calculated for hFMO3 wild type and N61S variant.

	Reduction		Re-oxidation	Intermediate	
	*K*_*d*_,NADPH	*k*_*red*_	*k*_*ox*_	*k*_*formation*_	*k*_*decay*_
	*(μM)*	* (s* ^*−*1^ *)*	* (s* ^*−*1^ *)*	* (s* ^*−1*^ *)*	* (s* ^*−1*^ *)*
Wild type	0.3	1.33	*R*_*1*_ = 0.79 *R*_2_ = 0.0009	1.580	0.001
N61S	51.8	ND	0.70	0.332	0.048
		2.42^#^			

ND: not detected.

^#^10-fold higher concentration of NADPH.

**Figure 1:**
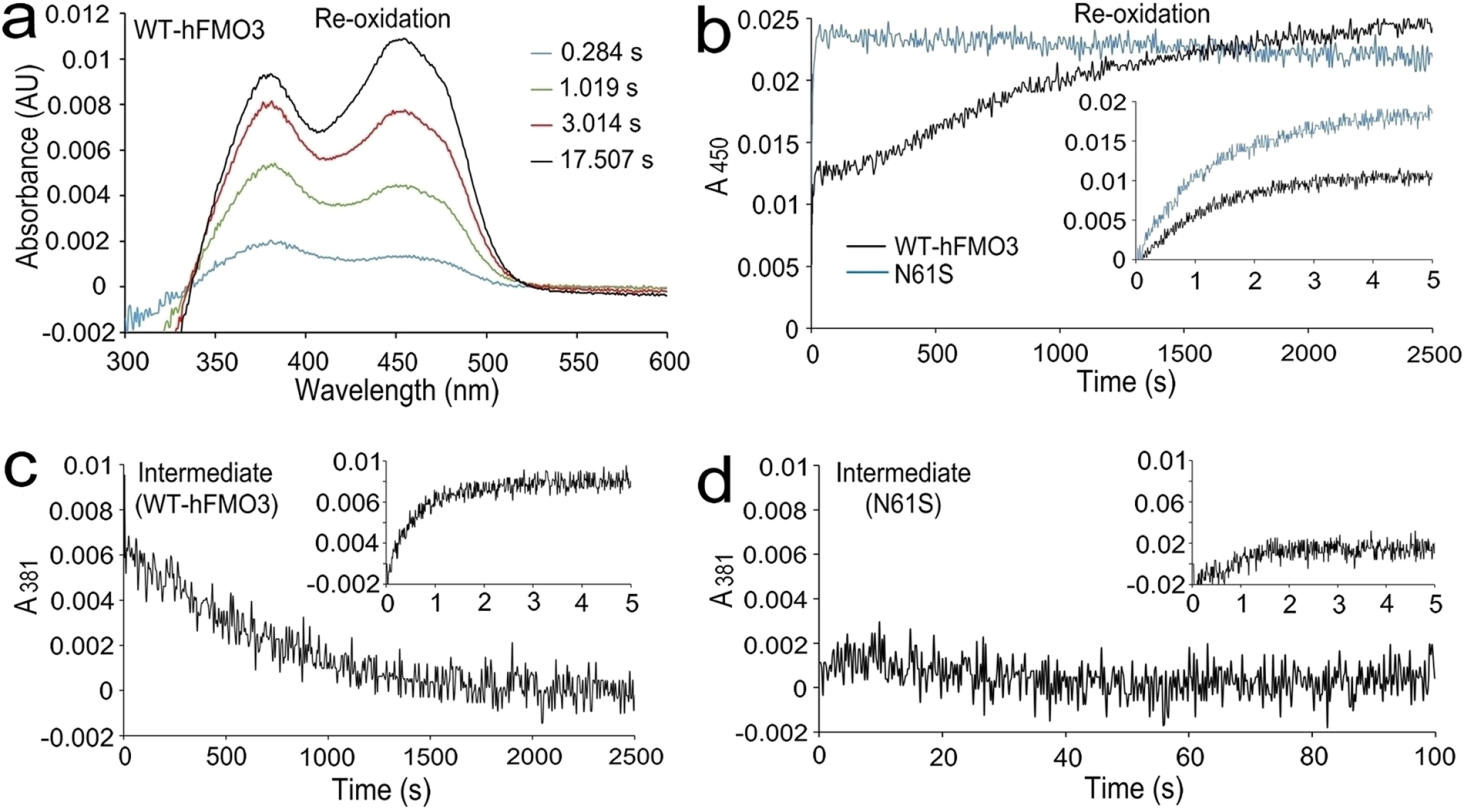
(**a**) The re-oxidation spectra of reduced WT-hFMO3 at four different time points. (**b**) Re-oxidation processes of WT-hFMO3 (black line) and N61S variant (blue line) followed at 450 nm over time. Formation and decay of the C4a-hydroperoxyFAD intermediate followed at 381 nm; (**c**) WT-hFMO3 and (**d**) N61S variant. The figure insets show the formation of the C4a-hydroperoxyFAD intermediate followed at 381 nm during the first five seconds.

The fully reduced protein was used to investigate the oxidative half-reaction after mixing with air-saturated buffer in the stopped-flow apparatus and by monitoring the FAD absorbance spectra within 300–600 nm. For clarity, only a few spectra of the WT-hFMO3 are shown in Fig. 1A. Using these re-oxidation spectra, the increase in FAD absorbance at 450 nm was plotted against time (Fig. 1B). As can been seen in Fig. 1B, the reduced WT-hFMO3 was fully re-oxidized after around 2300 seconds, but for N61S variant no further re-oxidation was observed after 30 seconds, suggesting that the mutation accelerated the re-oxidation process. The kinetics data of WT-hFMO3 re-oxidation were best fitted to a two-step process with a rate constant of 0.79 s^−1^ for the first step and 0.0009 s^−1^ for the second step. On the other hand, the re-oxidation of the N61S variant was a one-step process with a calculated rate constant of 0.70 s^−1^ (Table 1).

It is known that the most crucial feature of the FMO catalytic cycle is the ability of the enzyme to stabilize the C4a-hydroperoxyFAD intermediate until a substrate gains access to its catalytic site^4,5^. Some FMOs form fairly stable C4a-hydroperoxyFAD intermediates whilst others have short-lived intermediates which in the absence of a substrate can be uncoupled and result in H_2_O_2_ production^22–24^. To investigate the formation and decay of C4a-hydroperoxyFAD intermediate, both reduced enzymes were mixed with oxygen in the stopped-flow. The C4a-hydroperoxyFAD intermediate was observed at 381 nm (Figure S1) which is within the spectral range reported for other flavin monooxygenases^25–27^.

Figure 1C and D shows the formation and decay of C4a-hydroperoxyFAD intermediate of WT-hFMO3 and N61S variant, respectively. For the WT-hFMO3, the intermediate is observed within the first five seconds (inset of Fig. 1C) and totally decayed after 2000 s. While for N61S variant (Fig. 1D), not only very little intermediate was observed but it also decayed very rapidly within 40 seconds. The rates of the formation and decay of this intermediate measured for the two enzymes are shown in Table 1. As can be seen in the latter table, the formation of the intermediate of the WT-hFMO3 was 5 times faster than that of the N61S variant (1.6 versus 0.3 s^−1^). Moreover, the intermediate of the N61S variant decayed much faster than that of the WT-hFMO3 (0.048 versus 0.001 s^−1^) suggesting a highly unstable intermediate. Taken together, the stopped-flow data analysis signifies that the N61S mutation destabilizes the C4a-hydroperoxyFAD intermediate and accelerates its decay process.

### Circular dichroism studies

**Figure 2:**
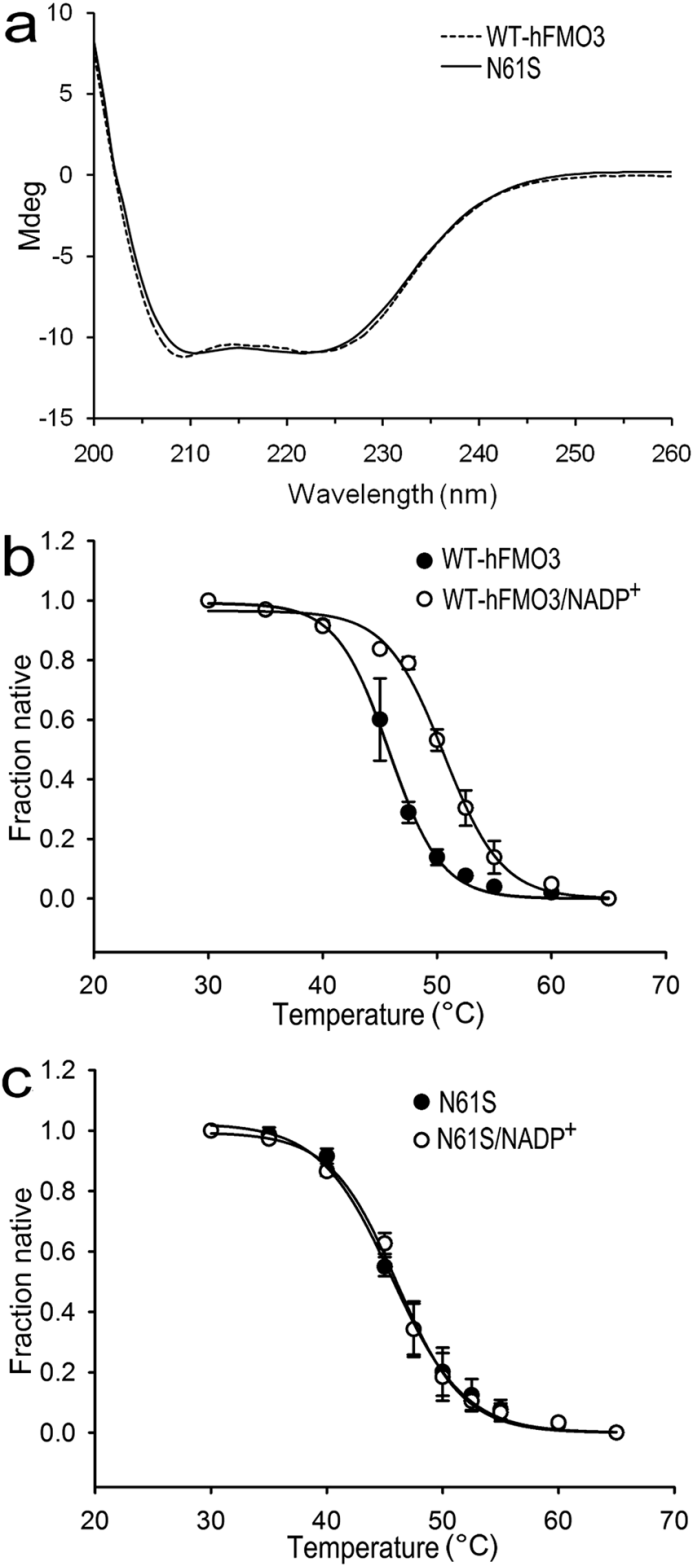
(**a**) Superimposition of CD spectra of WT-hFMO3 (dash line) and its N61S variant (solid line). Thermal unfolding profiles of (**b**) WT-hFMO3 and (**c**) N61S variant monitored at 222 nm (without NADP^+^, closed circle; with NADP^+^, open circle).

As an initial starting point for investigating the reasons behind the destabilisation of the N61S intermediate, CD experiments were carried out. The CD spectra of WT-hFMO3 and N61S measured at 30 °C show minima at 208 and 222 nm, which is consistent with an alpha-helical protein containing around 40% helical conformation as reported before^28,29^. The superimposed far-UV CD spectra of WT-hFMO3 and N61S are shown in Fig. 2A and demonstrate that the mutation does not affect the overall fold of the protein but small local conformational change(s) cannot be excluded.

Since the 2D structure of the variant was seen to be nearly identical to the WT-hFMO3, the effect of NADP^+^ binding to the two proteins was investigated in order to see whether the binding of this co-substrate has any stabilisation effect. To this end, the thermal unfolding of the proteins in the absence and presence of NADP^+^ were evaluated by monitoring the ellipticity at 222 nm from 30 °C to 75 °C. The melting temperature (T_m_) of the proteins was subsequently determined by fitting the ellipticity versus temperature to sigmoidal transitions (Fig. 2B-C) suggesting the proteins’ unfolding occurred in a two-state manner without accumulation of any unfolding intermediates. The resulting data are summarised in Table 2. The T_m_ for WT-hFMO3 and N61S variant in the absence of NADP^+^ was 45.8 and 45.6 °C, respectively, indicating that the mutation did not significantly change the overall structural stability of the protein. However, in the presence of NADP^+^, the T_m_ for WT-hFMO3 increased by nearly 5 °C to 50.6 °C. On the other hand, the measured T_m_ for the N61S variant remained nearly the same in the presence or absence of NADP^+^, around 46.0 °C (Fig. 2C and Table 2), suggesting that NADP^+^ did not bind or impart structural stability to the protein. This CD data are in line with the observed fast decay of the C4a-hydroperoxyFAD intermediate discussed above.

**Table 2:** Melting temperatures of WT-hFMO3 and N61S variant determined by CD.

Ligand	Melting temperature (°C)	
	WT-hFMO3	N61S
Without NADP^+^	45.80 ± 0.04	45.62 ± 0.04^#^
With NADP^+^	50.62 ± 0.17*	46.05 ± 0.25**^##^

^*^P < 0.001, **P < 0.01 compared to no NADP^+^; ^#^P > 0.05, ^##^P < 0.001 compared to WT-hFMO3, two-way ANOVA followed by Student-Newman-Keuls test.

### Isothermal Titration Calorimetry (ITC)

**Figure 3:**
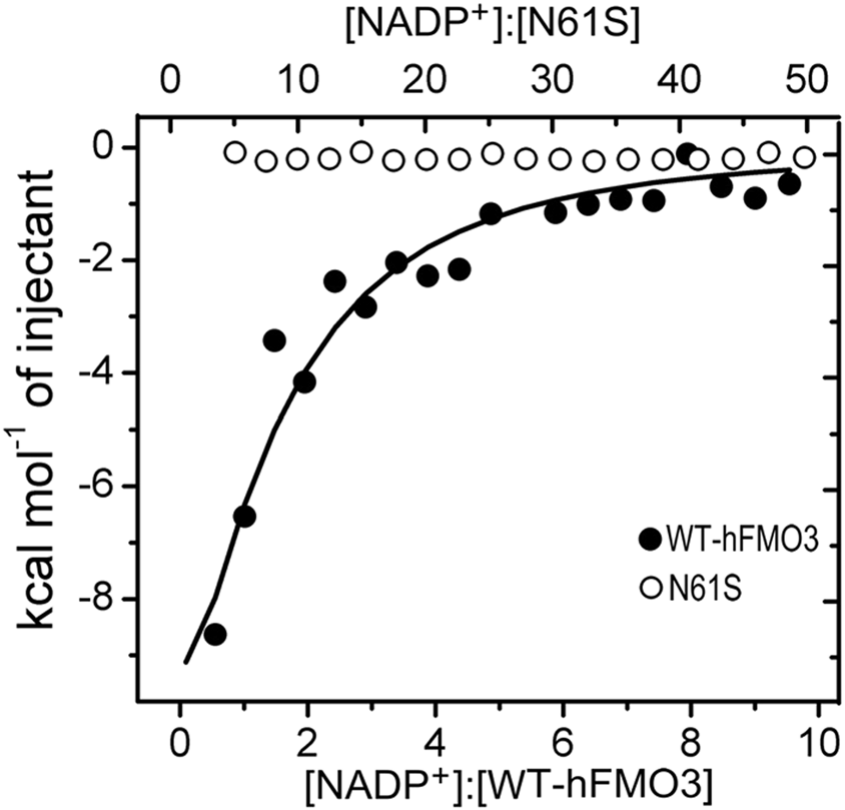
NADP^+^ binding profiles for WT-hFMO3 (close circle) and N61S variant (open circle). The titration for N61S was carried out at a higher ligand-to-protein ratio (20 μM N61S titrated with 8 mM NADP^+^) than the titration for WT-hFMO3 (20 μM titrated with 1.5 mM NADP^+^) due to its poor NADP^+^ binding affinity.

It has been previously suggested that the conserved asparagine residue, N61, may play a critical role in the stabilization of NADP^+^ binding to the protein^18^. In addition, the CD data demonstrate that the presence of NADP^+^ has no effect on the stability of the N61S variant. Therefore, binding affinities of WT-hFMO3 and N61S variant for NADP^+^ were measured by ITC^30^. The data are shown in Fig. 3 where the integrated heat versus NADP^+^/WT-hFMO3 molar ratio for each injection point was normalized and resulting in a sigmoidal binding curve. This curve was fitted to a single-site binding mode (n = 1 binding site) with a calculated dissociation constant of K_d_ = 3.7 μM.

The same ITC experiments were carried out with the N61S variant but in this case no appreciable binding of NADP^+^ could be measured even at higher ligand-to-protein ratios (control titration experiments are shown in Figure S2). As can be seen in Fig. 3, the N61S variant either does not bind NADP^+^ or its binding affinity is too low to be quantified by ITC, which is consistent with the CD results.

### Differential scanning calorimetry (DSC)

To further investigate the NADP^+^ binding properties, the transition midpoint (T_m_) of WT-hFMO3 and N61S variant in the absence and presence of NADP^+^ was determined by DSC. As an initial experiment the reversibility of the unfolding of WT-hFMO3 was studied but the rescanned DSC thermographs revealed that the hFMO3 unfolding is irreversible (Fig. S3).

**Figure 4:**
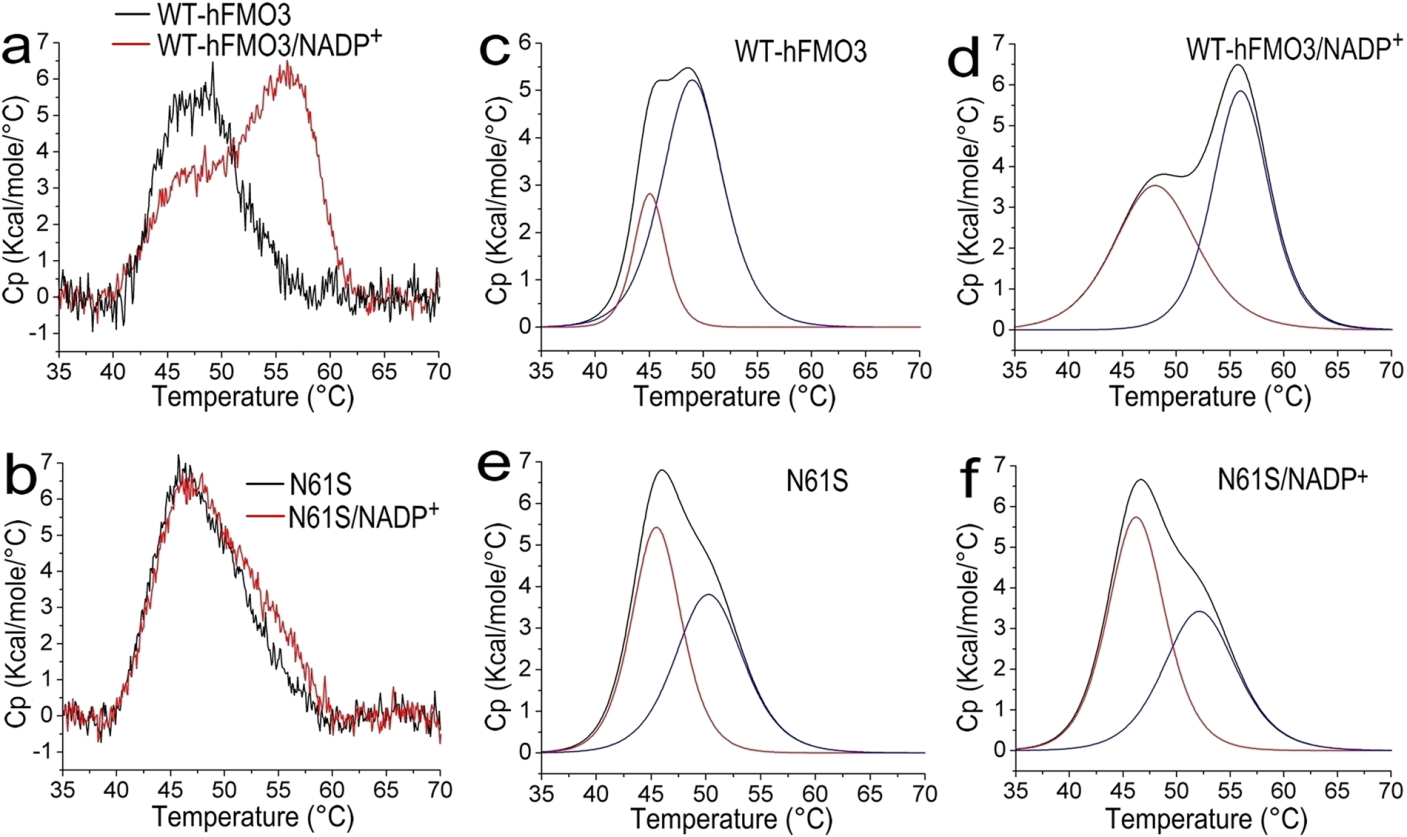
DSC data for WT-hFMO3 (**a**) and N61S variant (**b**) in the absence (black line) and presence (red line) of 0.5 mM NADP^+^, upon heating at a scan rate of 30 °C/h. Deconvolution of the DSC profiles: (**c**) WT-hFMO3; (**d**) WT-hFMO3 incubated in 0.5 mM NADP^+^; (**e**) N61S variant; (**f**) N61S variant incubated in 0.5 mM NADP^+^. The original scan is shown in black with the deconvoluted curves shown in red and blue.

For the DSC experiments, each protein was placed with the cell and the temperature increased from 35 °C to 70 °C. In order to maximize the resolution of the experiments, all were performed at the low scan rate of 30 °C/h, as mentioned in the Materials and methods section. The results obtained for the temperature dependence of the excess heat capacity for each of the two proteins are shown in Fig. 4. As can be seen in Fig. 4, a single symmetrical peak was not observed suggesting that the thermal unfolding of hFMO3 and its N61S variant is not a simple two-state process^31^ with or without NADP^+^. Furthermore, for both proteins in the absence of NADP^+^ and at 50 °C a shoulder appears near the main peak of DSC thermograph (centred around E °C) (Fig. 4C and E). A similar trend is also observed in the data in the presence of NADP^+^ but at different temperatures (Fig. 4D and F). This observation indicates the existence of two underlying melting processes which may be related to two different domains (NADP^+^ binding domain and FAD binding domain) of hFMO3 molecule^19^, or the presence of two protein populations (native plus aggregated forms).

The original DSC curves were therefore deconvoluted (Fig. 4C,D,E, and F) in order to calculate the T_m_ of the unfolding process for each of the two proteins in the presence and absence of NADP^+^. The data are summarised in Table 3 where for the WT-hFMO3 significant increases in T_m_ are observed upon NADP^+^ binding (3 °C for T_m1_ and 7 °C for T_m2_). On the other hand, for N61S variant no obvious peak shift is observed in the absence or presence of NADP^+^ (only 0.7 °C for T_m1_ and 1.9 °C for T_m2_) again indicating the poor NADP^+^ binding affinity which is also in line with the CD and ITC data.

**Table 3:** The melting temperatures for WT-hFMO3 and N61S measured by ITC in the presence and absence of NADP^+.^

protein	T_m1_, °C	T_m2_, °C
WT-hFMO3	45.1 ± 0.1	49.0 ± 0.2
WT-hFMO3/NADP^+^	48.1 ± 0.2*	56.0 ± 0.1*
N61S	45.5 ± 0.1^#^	50.3 ± 0.2^##^
N61S/NADP^+^	46.2 ± 0.1*^,##^	52.2 ± 0.2*^,##^

^*^P < 0.001 compared to no NADP^+^; ^#^P < 0.01^, ##^P < 0.001 compared to WT-hFMO3, two-way ANOVA followed by Student-Newman-Keuls test.

### Trypsin digestion studies

The data presented up to this point corroborate the fact that the WT-hFMO3 and N61S variant behave differently in the presence of NADP^+^: the presence and binding of this co-substrate has a stabilising effect only on the WT-hFMO3. We further investigated the effect of NADP^+^ binding by limited trypsin proteolysis which, in the absence of a crystal structure, could be a useful indication of exposure to digestion consequent to protein folding and suggest regions of highest flexibility^19,32^.

**Figure 5:**
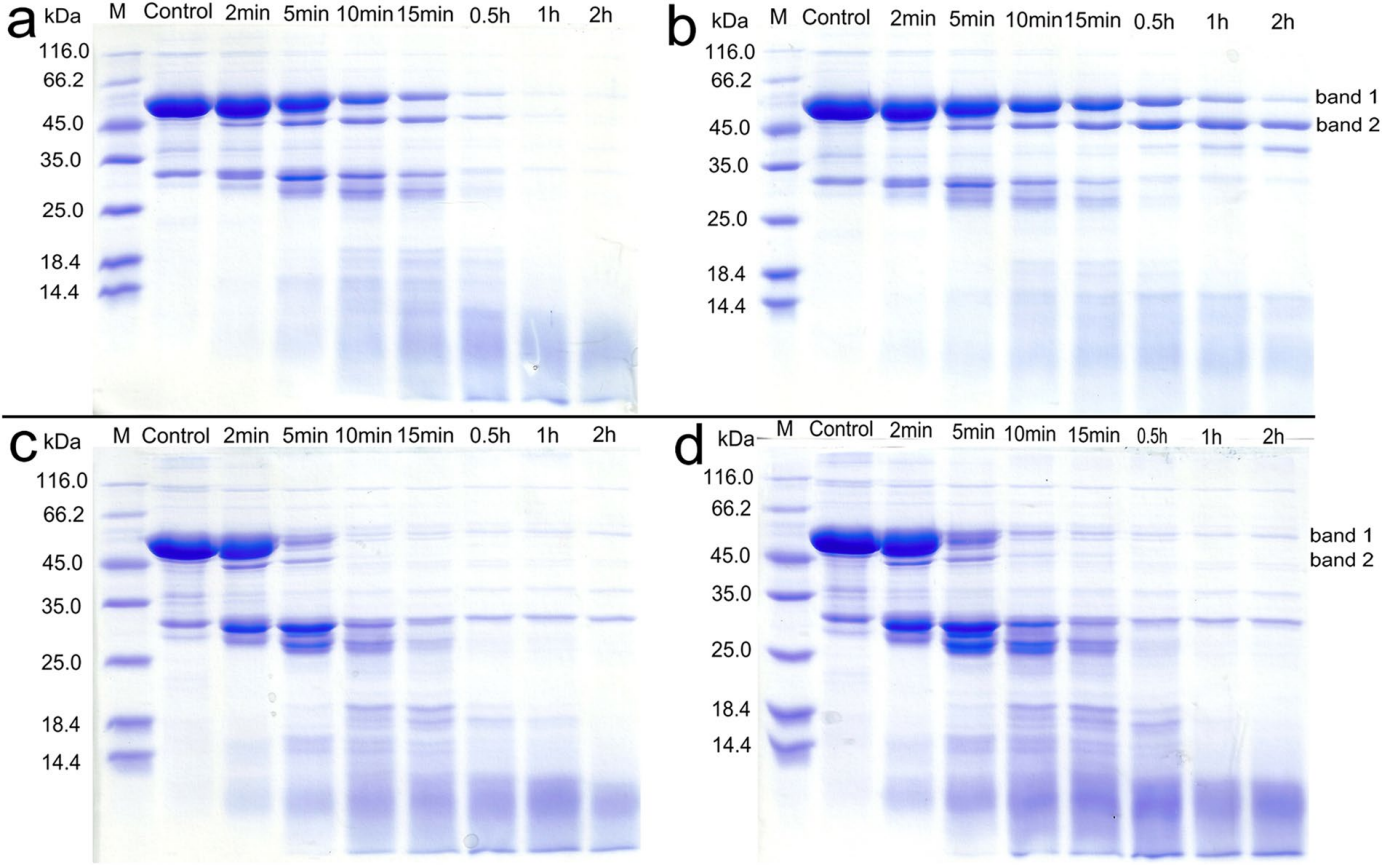
Trypsin digestion of WT-hFMO3 (Top panel) and N61S variant (Bottom panel) in the absence (**a** and **c**) and presence (**b** and **d**) of NADP^+^. Protein concentration was 1.45 μg/μl digested with trypsin (0.75 μg/μl) at 37 °C.

As can be seen in Fig. 5, the trypsin digestion results of WT-hFMO3 and its N61S variant in the absence of NADP^+^ already show differences (Fig. 5A and C). The first observation is that the major protein band at 58 kDa, corresponding to hFMO3, is already degraded within the first 5–10 min of the N61S digestion whereas the same pattern appears in the WT-hFMO3 gel after 30 min (Fig. 5A and C). After statistical analysis, the differences observed in the degradation patterns of WT and N61S in absence of NADP^+^ (Fig. 5A and C) were found to be significant (P < 0.05). The CD data presented demonstrated that any differences were due to local conformational changes rather than secondary structure content. The second observation is that the presence of NADP^+^ protects the WT-hFMO3 from trypsin digestion with some protein at 58 kDa still present after 2 hours of digestion (Fig. 5B). Again, carrying out a statistical analysis on the two gels shown in Fig. 5A and B, the WT-hFMO3 digestion pattern was found to be significantly affected by the presence of NADP^+^ (P < 0.05). These changes in digestion band patterns may be due to the co-substrate binding resulting in a more compact/close structure of the protein with a decrease in the flexibility of the folded domains. On the other hand, minor differences were observed for N61S variant in the presence of NADP^+^ (Fig. 5D) with no statistical significance, consistent with CD, ITC and DSC data.

### Steady-state kinetic experiments

The data presented in the previous sections pointed to the fact that the N61S variant not only had a much lower affinity for NADPH but that the small amount of C4a-hydroperoxyFAD intermediate observed in the presence of oxygen, decayed rapidly. This leads to the question of whether the N61S variant is at all catalytically active in terms of substrate conversion. To this end, four different known substrates of hFMO3 were selected, namely TMA, methimazole, benzydamine and tamoxifen and the kinetic parameters for N- or S-oxygenation of each of these substrates was determined with the two enzymes. For benzydamine and tamoxifen the N-oxide products were separated and measured using HPLC.

The calculated kinetic parameters for the four different substrates are summarised in Table 4. As expected and consistent with previous reports^12,33^, the N61S mutation completely abolishes the catalytic activity of the enzyme towards TMA N-oxygenation leading to fish odour syndrome. For the other remaining substrates, the N61S variant was catalytically active although significant increases in the calculated *K*_*m*_ values (ranging from 3–580 folds) and lower specific activity compared to WT-hFMO3 were observed.

**Table 4:** Kinetic parameters for the oxygenation of four different substrates by WT-hFMO3 and N61S variant.

Substrate	Enzyme	*K*_*m*_ (μM)	*k*_*cat*_	*k*_*cat*_/*K*_*m*_
			(μM product/min/μM protein)	(min^−1^μM^−1^)
Trimethylamine	WT-hFMO3	21.7 ± 4.6	2.7 ± 0.2	0.12
	N61S	—	No activity	—
Methimazole	WT-hFMO3	14.9 ± 2.0	8.4 ± 0.9	0.56
	N61S	8652.1 ± 351.6***	7.1 ± 0.9	0.0008***
Benzydamine	WT-hFMO3	56.0 ± 8	9.0 ± 2^a^	0.16
	N61S	417.3 ± 74.6**	2.0 ± 0.2*	0.005*
Tamoxifen	WT-hFMO3	5.8 ± 1.1^b^	16.4 ± 0.6^b^	2.83
	N61S	14.8 ± 3.4*	0.44 ± 0.02***	0.03**

^a^ ^28^, ^b^ ^38^.

^*^P < 0.05, **P < 0.01, ***P < 0.001 compared to WT-hFMO3, one-way ANOVA followed by Student-Newman-Keuls test.

## Discussion

Naturally occurring N61S mutation in hFMO3 results in defective TMA N-oxygenation giving rise to the fish-odour syndrome^11^. To date, the deciphering of the molecular mechanism underlying the loss of function of this variant has been hampered not only because of difficulties in purifying this human membrane-bound protein in its holo-form for characterisation of its reductive and re-oxidative transient kinetics, but also due to lack of structural information. Based on our knowledge of the successful purification of the WT-hFMO3 in its holo-form^28^, in this work we purified the N61S mutant and characterised the different steps of the catalytic cycle and compared the data to those of the WT-hFMO3.

The pre-steady-state kinetic parameters for the reductive half-reaction of N61S variant demonstrate that this mutant binds NADPH with 170-fold lower affinity than the wild type. Moreover, the steady-state kinetic experiments in the absence of substrate also reveal a decreased NADPH binding affinity, suggesting the mutation makes hFMO3 quite inefficient in utilising NADPH for flavin reduction. While for the re-oxidative half-reaction of N61S variant, stopped-flow measurements point to the rapid decay of the C4a-hydroperoxyFAD intermediate, indicating an unstable intermediate caused by mutation.

A previous report on the bacterial FMO has indicated the crucial role of NADP^+^ in the stabilization of C4a-hydroperoxyFAD intermediate^18^. In order to confirm whether the rapid decay of the N61S intermediate is caused by the binding properties of NADP^+^, several more detailed analytical tools such as CD, ITC and DSC were used. The data confirm that the N61S mutation results in poor NADP^+^ binding affinity with consequent destabilisation of the intermediate allowing for its observed rapid decay.

**Figure 6:**
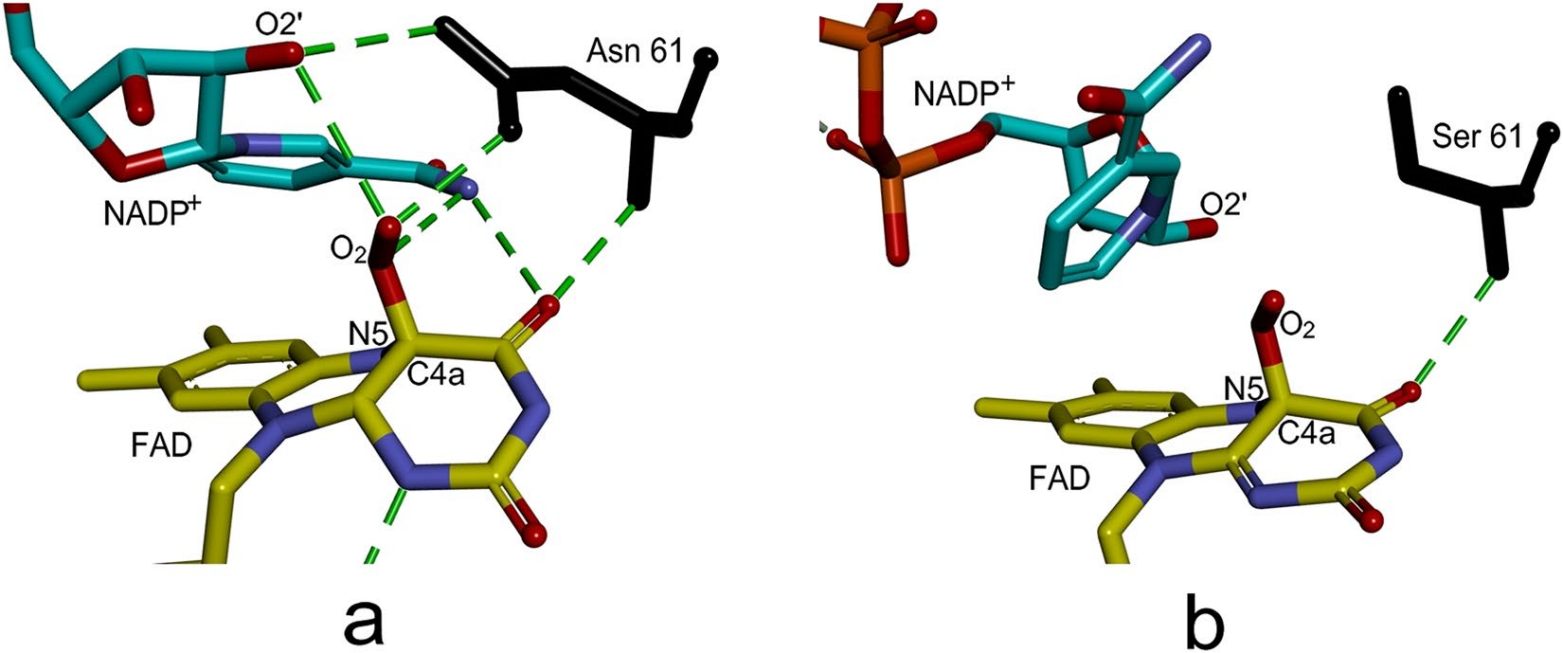
Molecular oxygen modelled at the C4a of FAD, followed by the docking of NADP^+^ to (**a**) hFMO3 model and (**b**) N61S variant. Docking performed by YASARA Structure package^19^. The hydrogen bond interactions are shown as green dots.

Structural studies on the yeast^17^ and the bacterial FMO^16^ have shown hydrogen bonding interactions between the conserved asparagine and NADP^+^, suggesting its important role in NADP^+^ binding. With the aid of the 3D homology model of hFMO3^19^, we demonstrate that these hydrogen bonds are also observed in NADP^+^/WT-hFMO3 molecular docking experiments as shown in Fig. 6A. Furthermore, the O2′ atom of NADP^+^ also forms hydrogen bonds with C4a-adduct of FAD in WT-hFMO3 model, also present in the bacterial FMO^16^ and, as a result stabilizes the oxygenating intermediate. On the contrary, the docking of NADP^+^ to N61S model results in a disordered binding mode with flavin N5 exposed, and no hydrogen bond between NADP^+^ and FAD/Ser61 (Fig. 6B). In the bacterial FMO, NADP^+^ prevents the decay of the C4a-hydroperoxyFAD intermediate by protecting the flavin N5 from solvent attack before substrate access^16^. Therefore, the poor NADP^+^ binding affinity of N61S variant, caused by the lack of the Asn61-NADP^+^ hydrogen bond, renders the flavin N5 solvent-exposed (Fig. 6B), which is unfavourable for the stability of its adjacent oxygenating intermediate. The molecular docking data of hFMO3 confirm the important role played by the conserved Asn61 in positioning and binding of NADP^+^ also in the human enzyme.

Finally, the steady-state kinetic experiments demonstrated that N61S mutation significantly affects hFMO3-mediated catalytic reactions, which is logical due to the instability of the intermediate which is detrimental for its oxygen-atom-transfer to the substrate. In a previous study^12^, it was suggested that this mutation only affects the hFMO3-mediated N- but not S-oxidation. However, the data presented here together with other reports^33^ clearly demonstrate the general negative effect this mutation has on the catalytic properties of the hFMO3.

In conclusion, this work provides details at molecular level on the impact of the N61S mutation on the activity of hFMO3. Individuals carrying this mutation will not only be inflicted by fish-odour syndrome but in addition will have problems in metabolising drugs which are substrates of hFMO3 since the data presented in this work demonstrate the instability of the most important intermediate within its catalytic cycle. More clinical studies in terms of drug metabolism and clearance need to be carried out in patients with fish-odour syndrome in order to fully understand the true extent of the problems caused by the loss of hFMO3 activity. Finally, it will be also interesting to see if individuals carrying this N61S mutation are naturally more resistant to diabetes and atherosclerosis due to absence of TMAO.

## Materials and Methods

### Cloning, expression and purification

The recombinant plasmids of pJL2-hFMO3 were constructed previously and the proteins expressed in *E*. *coli JM*1*09* cells^20,28^. Site-directed mutagenesis of hFMO3 was performed using QuikChange^®^ Site-Directed Mutagenesis Kit purchased from Stratagene. The kit employs PfuTurbo DNA polymerase to replicate both plasmid strands with high fidelity. The pJL2-hFMO3 recombinant plasmids were used as templates and the genes were amplified with the mutant oligonucleotide primers: 5′-GTCTTTTCCTCCTCTTCCAAAGAGATGATGTGTTTCCCA-3′ as forward, 5′-TGGGAAACACATCATCTCTTTGGAAGAGGAGGAAAAGAC-3′ as reverse. The mutation was confirmed by DNA sequencing.

Wild type and N61S mutant were expressed in *E*. *coli JM*1*09* at 24 °C for 24 hrs post-induction. Cell pellets were collected after centrifugation (4000 g, 20 min at 4 °C), followed by lysozyme treatment and ultra-sonication. The protein was extracted from membrane fractions using 1% IGEPAL and purified by a DEAE ion exchange column followed by Ni-affinity chromatography with buffers containing 0.1% IGEPAL. Eluted fractions containing FAD detected by a diode array HP-8453E spectrophotometer were collected and exchanged to storage buffer (100 mM phosphate buffer at pH 7.4, 20% glycerol and 1 mM EDTA) by 30 kDa cutoff Amicon membranes and stored at −80 °C. The presence and purity of the protein was confirmed by 12.5% SDS-PAGE. Protein concentration was determined spectroscopically as described previously^28^.

### Transient kinetics of reductive and oxidative half-reactions

The reduction by NADPH and subsequent oxidation by O_2_ of hFMO3 and N61S variant were monitored using a stopped-flow apparatus (SF-61, HI-TECH scientific) equipped with a diode array detector. All experiments were performed at 15 °C in an anaerobic glove box, and traces of oxygen in stopped-flow system were removed by washing the system using 0.1 M sodium acetate containing 18 U/ml glucose oxidase and 100 mM glucose, pH 5.0. For the reductive half-reaction, the protein (20 μM) in 50 mM phosphate buffer (pH 7.4) was first made anaerobic in the glove box and loaded into one drive syringe. NADPH dissolved in the same buffer was also made anaerobic and loaded into the other drive syringe. The reaction was started and monitored over time after the solutions were injected from the drive syringes into a mixing chamber (1:1 v/v). Spectral data (300–700 nm) of reductive half-reactions performed at various concentrations of NADPH were collected and analysed by Kinetic Studio Version 3.0 software package. The rates of reduction at different concentrations of NADPH were determined by fitting the decreased absorbance at 450 nm to a single exponential decay equation (1). The observed reaction rates with increased NADPH concentrations from at least 3 separate experiments could be fitted quite well with equation 2 using non-linear regression analysis (SigmaPlot 12.0 for windows).

1 $${A}_{450}=-B\times {e}^{(-R\times t)}+C$$

2 $${k}_{obs}=\frac{{k}_{{\rm{red}}}[{\rm{NADPH}}]}{{K}_{{\rm{d}}}+[{\rm{NADPH}}]}$$

In order to follow the re-oxidation of the reduced hFMO3 and N61S variant, each protein solution was mixed with equimolar amounts of NADPH under anaerobiosis to full reduction. The reduced protein was subsequently mixed with air-saturated buffer and the formation of oxidized FAD followed by the increase in absorbance at 450 nm. The re-oxidation data were analysed by Kinetic Studio Version 3.0 software package.

### Circular dichroism

The far-UV CD spectra of WT-hFMO3 and its N61S variant were determined by a J-815 CD spectrometer (JASCO International Co., Tokyo, Japan) with protein concentrations between 0.2 mg/ml and 0.25 mg/ml in 50 mM phosphate buffer pH 7.4, in the absence and presence of 0.5 mM NADP^+^. Spectra were recorded from 190 nm to 260 nm at a scan rate of 200 nm/min and 0.5 nm data pitch using 1 nm band width, with the temperature regulated using a PTC-423S heat control unit (JASCO). Ten accumulations were recorded and averaged for each spectrum using a 0.1 cm path length cell scanned at 30 °C, 35 °C, 40 °C, 45 °C, 47.5 °C, 50 °C, 52.5 °C, 55 °C, 60 °C and 65 °C. The results in this study were the mean of at least 3 different measurements. Thermal unfolding profiles and CD data were analysed as previously described^18,34^.

### Isothermal titration calorimetry (ITC)

ITC experiments were performed using the ITC_200_ calorimeter to measure enthalpies of the reaction at 25 °C. An initial 0.4 μl of NADP^+^ solution with a known concentration was injected into the sample cell containing 280 μl protein (20 μM), followed by 18 × 2 μl injections. The initial 0.4 μl injection was removed for clarity. Each injection lasted 4 s with an interval of 150 s between injections, and the protein solution in the sample cell was stirred at a speed of 750 rpm. All solutions were thoroughly degassed prior to loading in the syringe and sample cell.

The heat evolved for each titration is due to both the binding of ligand to receptor and the dilution of ligand solution. In order to determine the heat of binding, the heat of dilution was subtracted from the experimental data. The heat of dilution was determined by a second titration in which no protein was in the cell while keeping all the other experimental parameters constant. After correction for heats of dilution, the integrated heat effects were analysed by nonlinear regression using “One Set of Sites” curve fitting model (MicroCal ITC_200_ Origin). The fitted data yield the association constant (K_a_), reaction stoichiometry (n), enthalpy (*ΔH*) and entropy (*ΔS*) of binding. The dissociation constant (K_d_) can be calculated by the inverse of the association constant.

All data were collected at least in three different replicates and reported as mean ± standard deviation. Statistical relevance of the differences detected between the collected results was analysed by one-way ANOVA and two-way ANOVA followed by Student-Newman-Keuls post hoc test using SigmaPlot software.

### Differential scanning calorimetry

DSC experiments were carried out using a Microcal VP-DSC instrument with protein concentration of 10 μM at a scan rate of 30 °C/h. Samples were degassed extensively under vacuum before each experiment. Scans were preformed from 30 °C to 75 °C, then immediately cooled back to 30 °C and held for 10 min for equilibration and a second scan (rescan) was carried out at the same scan rate as the control. These scans were performed in 50 mM phosphate buffer pH 7.4, in the absence and presence of 0.5 mM NADP^+^. Prior to data analysis, the control (rescan data) was subtracted from the experimental data, followed by data normalization for protein concentration. Deconvolution analysis^35^ and plotting of the calorimetric data was performed using Origin software (MicroCal).

### Trypsin digestion analysis

Trypsin endopeptidase can cleave proteins at the carboxyl side of the solvent-accessible lysine and arginine residues making it useful in the measurement of protein folding topology^19,32^. To investigate the conformational change upon NADP^+^ binding, hFMO3 and its N61S variant were digested with trypsin and the band patterns produced over time were analysed by SDS-PAGE. Briefly, 116 μg protein in 80 μl phosphate buffer (pH 7.4), in the absence and presence of 2.5 mM NADP^+^, was mixed with 60 μg trypsin and incubated for 2, 5, 10, 15, 30, 60, 120 min at 37 °C. At each time interval, 10 μl of the reaction mixture was removed and mixed with 10 μl Laemmli (60 mM Tris-HCl pH 6.8/ 2% SDS/ 12% glycerol/ 0.001% bromophenol blue), then immediately heated to 98 °C for 10 min. All samples collected in this manner were subsequently subjected to 12.5% SDS-PAGE analysis.

The bands on the SDS gels were quantified by ImageJ software (http://rsb.info.nih.gov/ij/). Statistical relevance of the differences detected between the two major bands of the SDS-PAGE gels were carried out by repeated-measures two-way ANOVA followed by Student-Newman-Keuls post hoc test using SigmaPlot software.

### Enzyme assays

Kinetic parameters for WT-hFMO3 and N61S-mediated TMA N-oxygenation were determined spectrophotometrically by monitoring the oxidation of NADPH at 340 nm (molar extinction coefficient: 6.22 mM^−1^ cm^−1^). The reaction mixture contained 50 mM phosphate buffer pH 7.4, 0.2 mM NADPH and 0.6 μM enzyme. Reactions were initiated by adding different amounts of TMA and performed at 37 °C for 10 minutes. The final TMA concentrations were 0.01, 0.025, 0.05, 0.1, 0.2, 0.4 mM for WT-hFMO3, and 0.05, 0.1, 0.2, 0.4, 1, 2, 5 mM for N61S variant. The control was performed in the absence of the substrate while keeping all the other experimental parameters constant.

Turnover of three other known substrates of hFMO3 were also carried out using the purified wild type and the N61S variant in order to determine the effect of this mutation on the activity of the enzyme. Methimazole S-oxygenation was followed spectrophotometrically via the reaction of the oxidized product with nitro-5-thiobenzoate (TNB) to generate 5,5′-dithiobis (2-nitrobenzoate) (DTNB)^36,37^. The reaction mixture contained 50 mM phosphate buffer pH 7.4, 60 μM DTNB, 30 μM DTT, 0.82 μM enzyme, different concentrations of methimazole and 1 mM NADPH. DTNB was initially reduced to TNB upon incubation with DTT for 5 minutes at 37 °C. Reactions were initiated by the addition of NADPH and performed at 37 °C for 10 minutes. The final methimazole concentrations were 0.01, 0.025, 0.05, 0.1, 0.2, 0.4, 1 mM for WT-hFMO3 and 0.2, 0.6, 1, 2, 4, 10, 20, 30 mM for N61S variant. The disappearance of the yellow colour was monitored spectrophotometrically at 412 nm and the specific activity (μM product/min/μM protein) was determined using the molar extinction coefficient of TNB (14.15 mM^−1^ cm^−1^).

N-oxygenation of benzydamine and tamoxifen by the wild type and N61S variant of hFMO3 were carried out as previously described^38,39^ and the amount of product determined by HPLC (Agilent-1200, Agilent Technologies, U.S.A.). For benzydamine N-oxygenation, the reaction mixture consisted of 0.3 μM enzyme, 0.5 mM NADPH, substrate (0–0.64 mM) in a final volume of 200 μl (50 mM phosphate buffer pH 7.4). While for tamoxifen N-oxygenation, the incubation mixture contained 0.5 mM NADPH, 2 μM enzyme and tamoxifen (0–160 μM). Incubations were performed at 37 °C for 10 min terminated by the addition of 100 μl of ice-cold acetonitrile and centrifuged at 14000 × g for 5 min. 100 μl of supernatant for each sample was analysed by HPLC equipped with 4.6 × 150 mm 5 μm Eclipse XDB-C18 column at room temperature with the UV–visible detector set at 308 nm for benzydamine N-oxide and 276 nm for tamoxifen N-oxide. The separation of benzydamine and its N-oxide a mobile phase of 30% acetonitrile and 70% 30 mM phosphate buffer pH 7.2 was used at a flow rate of 1.0 ml/min. For the separation of tamoxifen and its metabolite, a mobile phase of 82% methanol and 18% triethylamine (1%) at a flow rate of 1.0 ml/min was used.

The steady-state activity of WT-hFMO3 and N61S variant was also measured in the absence of substrate by monitoring the decrease of NADPH with time at 340 nm. The reaction mixture contained 50 mM phosphate buffer pH 7.4, 0.2 μM enzyme with various concentrations of NADPH. Reactions were carried out at 37 °C for 20 min.

All the measurements were performed in triplicates at minimum.

### Data Availability

All data generated or analysed during this study are included in this published article.

## Electronic supplementary material

supplementary info
