# Supplementary material for: Inactivation mechanism of N61S mutant of human FMO3 towards trimethylamine
Source: Sci Rep. 2017 Nov 7;7:14668. doi: 10.1038/s41598-017-15224-9 (PMC5676948; doi:10.1038/s41598-017-15224-9)
Supplement: Supplementary file 1 — supplementary info [file 41598_2017_15224_MOESM1_ESM.pdf]

SUPPLEMENTARY INFORMATION

Inactivation mechanism of N61S mutant of human FMO3 towards trimethylamine

Chongliang Gao, Gianluca Catucci, Silvia Castrignanò, Gianfranco Gilardi and Sheila J. Sadeghi*

Department of Life Sciences and Systems Biology, University of Torino, Italy

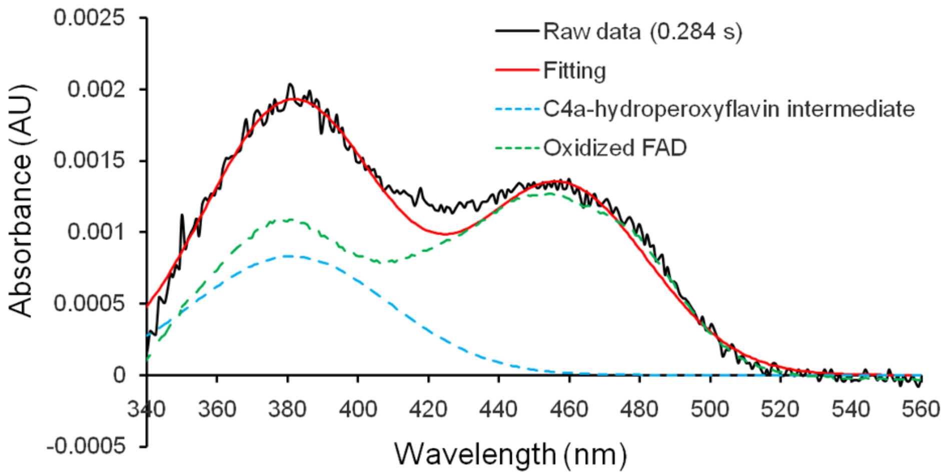

Figure S1

UV-vis spectrum of C4a-hydroperoxyflavin intermediate of hFMO3 with a maximum absorbance at 381 nm, observed by deconvolution of the single spectrum of the stopped-flow data shown in Fig. 1A (0.284 s).

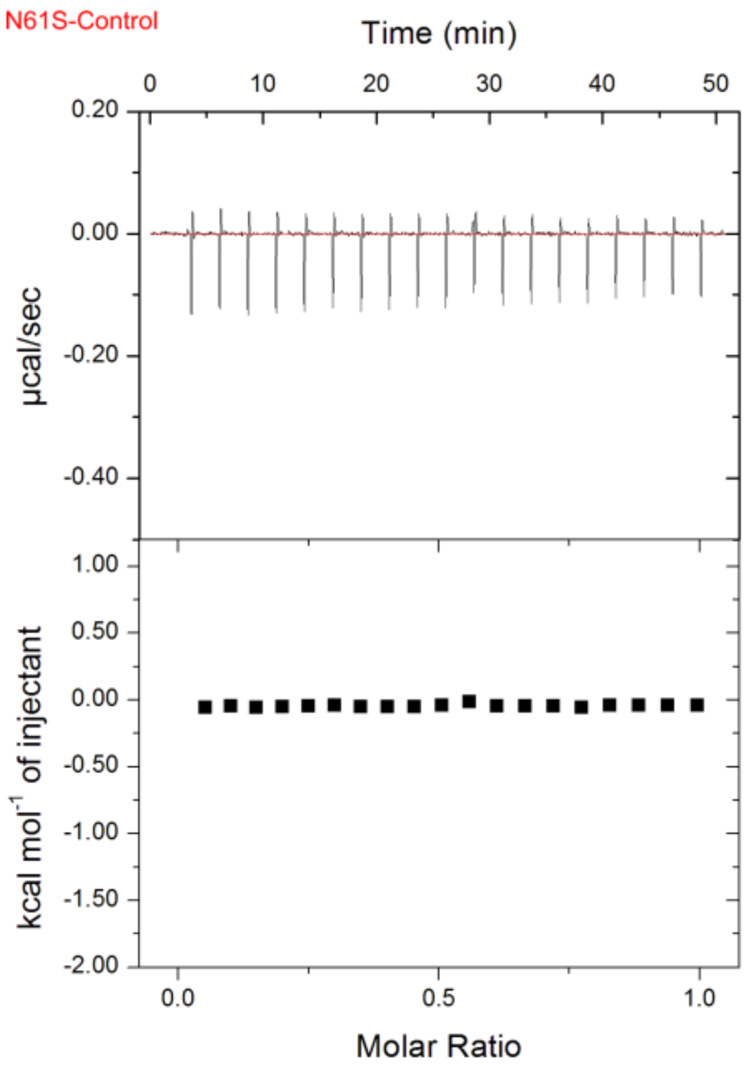

Figure S2
Control Isothermal Titration Calorimetry (ITC) experiments. The titration of N61S mutant was carried out with 8 mM $NADP^+$. For control experiment, the sample cell contained buffer and the ligand, $NADP^+$, was in the injector syringe.

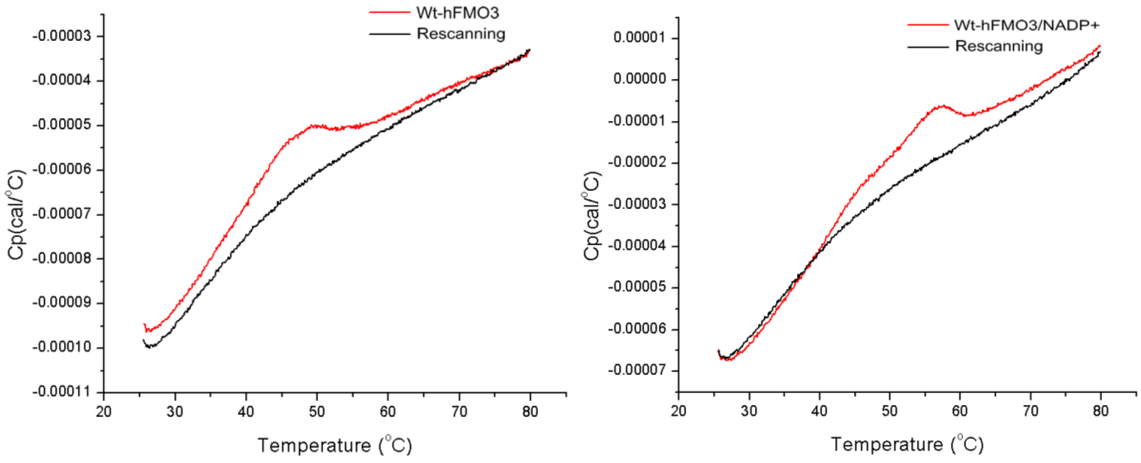

Figure S3

Differential scanning calorimetry (DSC). Human FMO3 unfolding is irreversible as shown by the rescanned DSC thermographs both in the absence (left) and presence (right) of $NADP^+$. Initial scans (red line) were performed from 30 °C to 75 °C and immediately cooled back to 30 °C and held for 10 min for equilibration. This was followed by a second scan, rescan (black line), with the same scan rate.

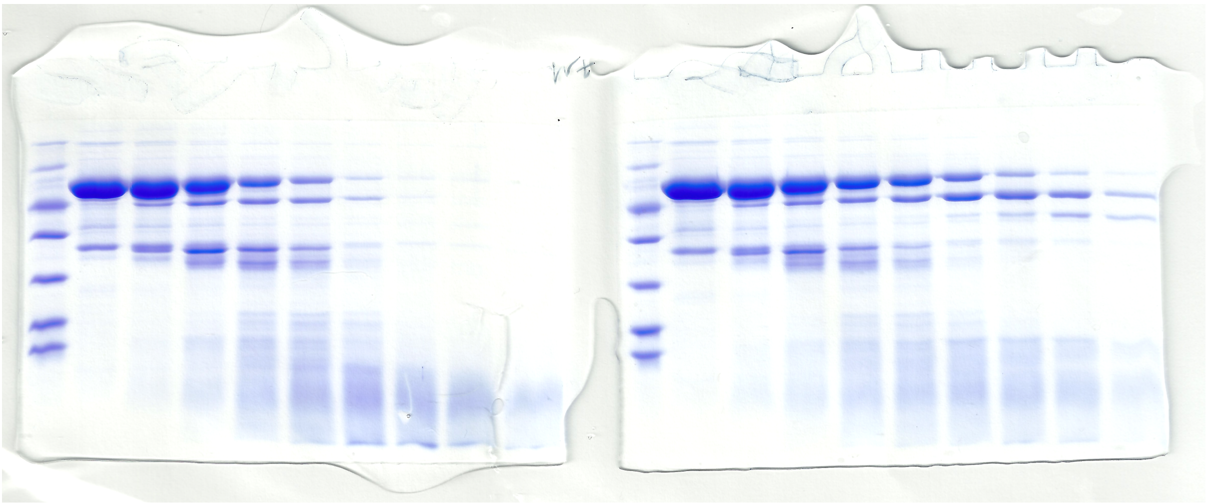

Figure S4

Scan of original SDS-PAGE gels of WT-hFMO3 trypsin digestion in the absence (left) and presence of NADP+ (right). Protein concentration was 1.45 µg/µl digested with 0.75 µg/µl of trypsin at 37 °C.

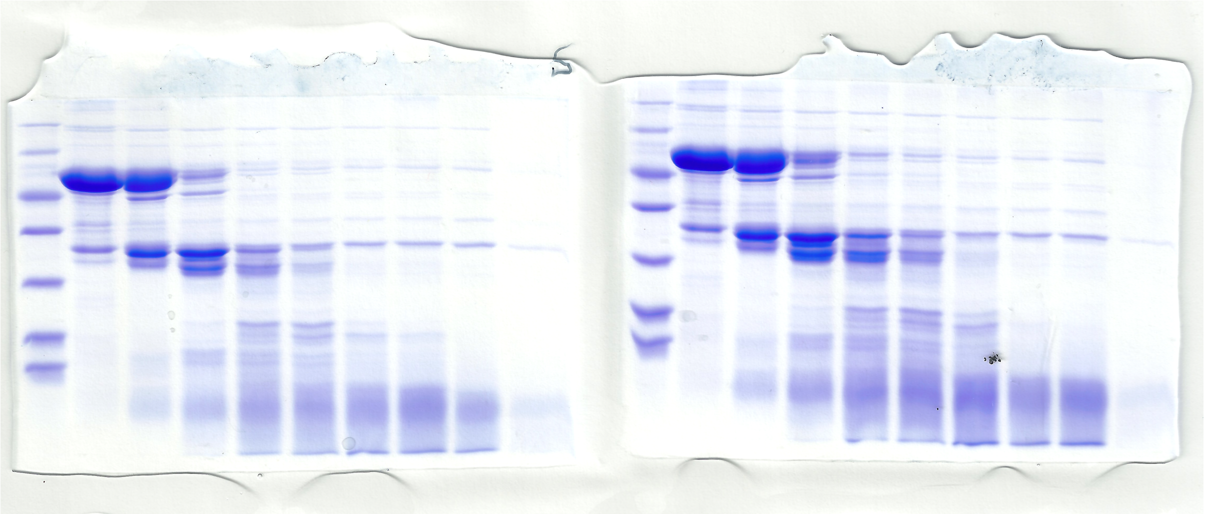

Figure S5
Scan of original SDS-PAGE gels of the trypsin digestion of N61S mutant in the absence (left) and presence of NADP+ (right). Protein concentration was 1.45 µg/µl digested with 0.75 µg/µl of trypsin at 37 °C.
